# Impaired brain network architecture in Cushing’s disease based on graph theoretical analysis

**DOI:** 10.18632/aging.102939

**Published:** 2020-03-24

**Authors:** Can-Xin Xu, Hong Jiang, Rui-Zhe Zheng, Yu-Hao Sun, Qing-Fang Sun, Liu-Guan Bian

**Affiliations:** 1 Department of Neurosurgery, Rui-Jin Hospital, Shanghai Jiao Tong University School of Medicine, Shanghai 200025, China; 2 Department of Neurosurgery, TongRen Hospital, Shanghai Jiao Tong University School of Medicine, Shanghai 200025, China; 3 Department of Neurosurgery, Rui-Jin Lu-Wan Hospital, Shanghai Jiao Tong University School of Medicine, Shanghai 200025, China

**Keywords:** Cushing's disease, brainnet, small world, rich club

## Abstract

To investigate the whole functional brain networks of active Cushing disease (CD) patients about topological parameters (small world and rich club et al.) and compared with healthy control (NC). Nineteen active CD patients and twenty-two healthy control subjects, matched in age, gender, and education, underwent resting-state fMRI. Graph theoretical analysis was used to calculate the functional brain network organizations for all participants, and those for active CD patients were compared for and NCs. Active CD patients revealed higher global efficiency, shortest path length and reduced cluster efficiency compared with healthy control. Additionally, small world organization was present in active CD patients but higher than healthy control. Moreover, rich club connections, feeder connections and local connections were significantly decreased in active CD patients. Functional network properties appeared to be disrupted in active CD patients compared with healthy control. Analyzing the changes that lead to abnormal network metrics will improve our understanding of the pathophysiological mechanisms underlying CD.

## INTRODUCTION

Cushing’s disease (CD), or pituitary-dependent Cushing's syndrome (CS) is a rare clinical syndrome, estimated incidence of 2.4 new cases per million inhabitants per year, and is characterized by excessive endogenous exposure to glucocorticoids (GCs), due to an adrenocorticotropic hormone (ACTH) secreting pituitary adenoma [1]. Patients with CD are exposed to high GC concentrations that stimulate the widely distributed mineralocorticoid (MR) and especially glucocorticoid (GR) receptors in the brain, causing abnormal alterations in brain structure and function. It has been conclusively shown that brain atrophy, abnormal changes in metabolism and white matter impairments in CD patients was caused by hypercortisolism [2–4]. These structural and functional changes in the brain can result in cognitive deficits, including poor visual memory and depression, in CD patients [5].

Human brain can be divided into distinct regions with different functions that form a whole-brain network system. Graph theory, a computational method, is an important tool to describe network characteristics. Nodes and edges are basic components of every brain network, with brain regions defined as nodes and connections between regions defined as edges, according to graph theory analysis. Graph theory analysis can transform networks into nodes, edges, thus making quantitative analysis of complex brain networks [6, 7]. Several studies have demonstrated that abnormal brain network organization compared with heathy control of neuropsychological disease and traumatic brain injury patients [8–10]. Parameters, such as global efficiency and local efficiency, are commonly used to reflect the strengths of brain network efficiency. The global efficiency of a network can quaintly reflect the ease of exchanging information over the whole network. Local efficiency is a network attribute that reflect how information is exchanged between the direct neighborhood of a node [11]. In recent years, small world and rich club organizations have been investigated in many diseases, and results have shown that understanding the brain network organizations may improve prognostication abilities and guide the development of new treatments in future [12]. In normal brain network there are shows more densely local connectivity and few long-rang connections, which is called small world organization. Brain hubs are regions that play vital roles during the integration of functional control and information flow throughout the whole network [13]. However, the brain connectivity and topologic alterations of the whole-brain connectome based on functional brain networks in CD patients have not yet been characterized. In recent years, advanced MRI has been greatly used to detect abnormal brain changes in CD patients [14]. For example, diffusion tensor imaging (DTI) [15], susceptibility-weighted imaging (SWI), especially functional MRI are all viable methods to detect abnormal brain connectives among brain regions that do not display obvious morphological changes [16]. Resting-state fMRI can not only detect abnormal functional connectivity but can also reflect the brain activity that occurs when a subject is not performing any specific task [14, 17, 18].

In this study, we used graph theory approaches to construct functional brain networks and further investigated the topological parameters of active CD patients compared with heathy control. We hypothesized the following: 1) active CD patients would be characterized by widespread network disruption; 2) the characteristics of small-world characteristic would be change in active CD patients based on functional brain networks; and 3) rich club organization may be disrupted in CD patients.

## RESULTS

### Demographic and clinical data

A total of 19 active CD patients and 22 healthy control (NC) were included for analysis. There were no significant differences in age (p=0.131) and gender (P=0.499) between active CD and controls (Table 1). Additionally, no significant differences were observed between the groups in terms of education. The disease duration of active CD patients was 1-15years (mean=4.76±3.68 years). active CD patients has significantly high 24H UFC (659.87±357.29ug/24h) and adrenocorticotropin levels (86.10±58.28 pg/ml) (Table 1). More detailed clinical information was shown in Table 1.

**Table 1:** Demographics and clinical data of participants.

	**Cushing Disease (n=19)**	**Controls (n=22)**	**P Value**
Age (y)	41.00±11.23	47.05±13.51	0.131^b^
Sex	4/15	7/15	0.499^a^
No. of Men	4	7	
No. of Women	15	15	
Education (y)	13.32±2.14	13.09±3.64	0.814^b^
Duration of illness (years)	4.76±3.58	-	-
Plasma Cortisol (0am) (ug/dl)	17.03±9.13	-	
Plasma Cortisol (4pm) (ug/dl)	19.66±9.09	-	
Plasma Cortisol (8am) (ug/dl)	2.43±13.08	-	
UFC_(21-111ug/24h)	659.87±357.29	-	
ACTH_ (7.0-65.0 pg/ml)	86.10±58.28	-	

Data are means and standard deviation unless otherwise noted. All of the scores are raw values. The comparisons of demographic between groups were performed with Mann-Whitney U test. P<0.05 indicated a significant difference. UFC: Urinary Free Cortisol; ACTH: adrenocorticotropin.

^a^Chi-squre test was used for calculated.

### Entire network analysis

**Figure 1:**
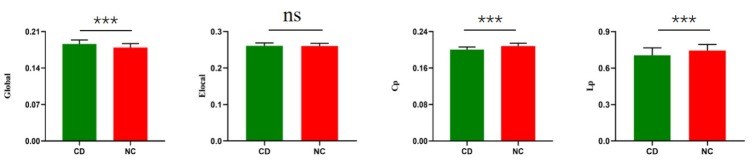
**Group differences between CD patients and healthy controls in the global of functional brain networks.** The bar and error bars represent the fitted values and standard deviations, respectively. Eglo= global efficiency, Eloc= local efficiency, Cp=cluster efficiency, Lp= shortest path length. CD= Cushing's disease, NC= healthy control.

In the range of 0.05<sparsity<0.40, global efficiency, local efficiency, clustering coefficients, shortest path length, small-world and rich club values for participants were calculated. Compared with NCs, the patients with active CD exhibited significantly increased network global efficiency (P = 0.002), shortest-path length (P = 0.026) (Figure 1). Compared with healthy control, active CD patients revealed significant decreased of cluster efficiency (P < 0.001). No significant difference in local efficiency was found between patients and NCs (P=0.223) (Figure 1).

### Small world

**Figure 2:**
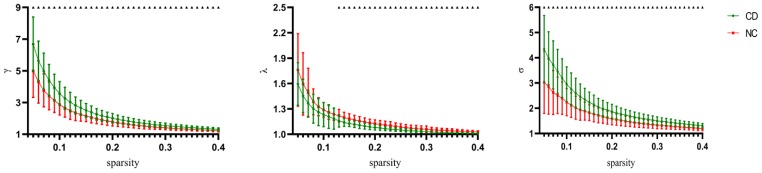
**Change of small world organization network definition parameters as parameters as a function of sparsity.** The error bars correspond to the standard error of the mean. Black triangle indicates points where the difference between the two groups is significant (P< 0.05, FDR corrected).

To clarify the small-world characteristics of functional brain network, we calculated the normalized clustering coefficient (γ), and the normalized characteristic path length (λ) of the brain network and compared them with those for corresponding random networks. In the range of 0.05 < sparsity < 0.40, we found that both CD patients and healthy control had small world properties (σ > 1) in functional brain networks (Figure 2) [false discovery rate [FDR]-corrected). However, active CD patients exhibited higher Sigma values over nearly the entire range of sparsity. The Lambda values of the active CD patients were lower than healthy control in most threshold ranges (Figure 2) (FDR-corrected). Compared with those for NCs, the γ values for active CD patients were significantly increased over sparsity ranging from 0.05 to 0.4 (Figure 2) (FDR-corrected).

### Rich club

**Figure 3:**
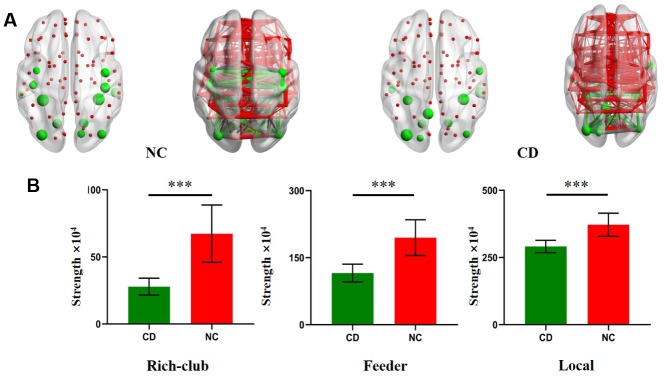
**Rich Club regions distributions in CD patients and NC.** (**A**) The hub nodes are shown with the node sizes indicating their nodal connection strength and rich club regions including the MTG.L, FFG.L, FFG.R, ITG.R, LING.L, LING.R, MOG.L, MOG.R, CUN.R, preCG.L, PreCG.R, PoCG.L, PoCG.R, SOG.L, PCUN.L, ITG.L, ROL.R. (**B**) The bar chart shows group differences in the rich-club, feeder, and local connection strengths. The bars and error bars represent the fitted values and the standard deviations, respectively.

In the NC group, multiple rich hubs were identified, including, MTG.L (left middle temporal gyrus), FFG.L (left fusiform gyrus), FFG.R (right fusiform gyrus), ITG.R (right inferior temporal gyrus), LING.L (left lingual gyrus), LING.R (right lingual gyrus), MOG.L (left middle occipital gyrus), MOG.R(left middle occipital gyrus), CUN.R (right cuneus), preCG.L (left precentral gyrus), PreCG.R (right precentral gyrus), PoCG.L (left postcentral gyrus), and PoCG.R (right postcentral gyrus) (Figure 3). In the active CD group, rich hubs regions were identified, including ITG.R(right inferior temporal gyrus), FFG.L (left fusiform gyrus), ^b^Mann-Whitney U test was used for calculated.

FFG.R (right fusiform gyrus), LING.R (right lingual gyrus), MOG.L (left middle occipital gyrus), MOG.R (right middle occipital gyrus), SOG.L (left superior occipital gyrus), PCUN.L (left precuneus), ITG.L (left, inferior temporal gyrus), and ROL.R (right rolandic operculum) (Figure 3).

For the further analysis, we calculated the connection strengths of rich-club connections, feeder connections and local connections of active CD patients and compared them with those of NCs. Compared with heathy control, rich club connections were significantly decreased in active CD patients. Additionally, significant reductions in local and feeder connections were found in active CD patients compared with NCs (Figure 3).

### Correlation analysis

**Figure 4:**
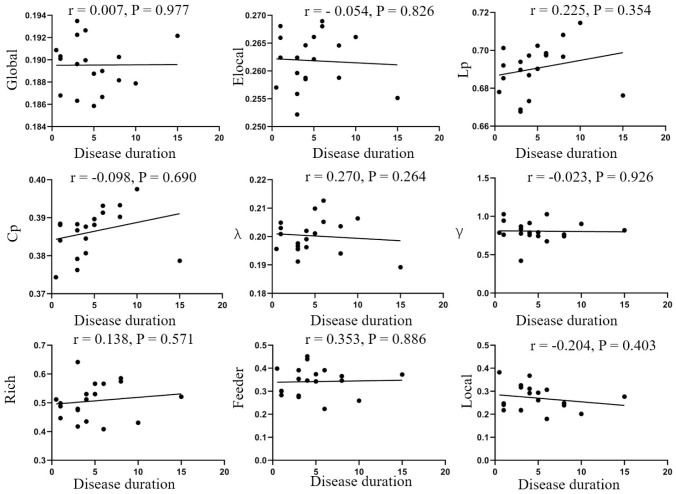
**Correlation analysis of disease duration and parameters of brain network.** No correlations were found in disease duration and global efficiency (r=0.007, p=0.977), local efficiency (r=-0.054, p=0.826), Lp (r=0.225, p=0.354), Cp (r=-0.098, p=0.690), λ (r=0.270, p=0.264), λ (r=-0.023, p=0.926), rich-club (r=0.138, p=0.571), feeder (r=0.353, p=0.886), local (r=-0.204, p=0.403). Elocal= local efficiency Cp=cluster efficiency, Lp= shortest path length.

**Figure 5:**
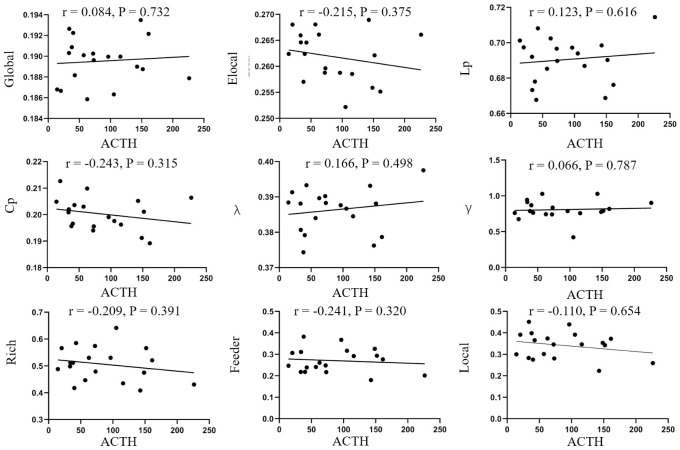
**Correlation analysis of ACTH and parameters of brain network.** No correlations were found in disease duration and global efficiency (r=0.084, p=0.732), local efficiency (r=-0.215, p=0.375), Lp (r=0.123, p=0.616), Cp (r=-0.243, p=0.315), λ (r=0.166, p=0.498), λ (r=-0.066, p=0.787), rich-club (r=-0.209, p=0.391), feeder (r=-0.241, p=0.320), local (r=-0.110, p=0.654). Elocal= local efficiency Cp=cluster efficiency, Lp= shortest path length.

No significant correlations between network parameters and disease duration were found (Figure 4). In addition, no significant differences were found between ACTH levels and the clinical information (Figure 5).

## DISCUSSION

In this study, we investigated functional brain networks, based on graph theory, and found abnormal changes of topological characteristics in active CD patients compared with NCs. To our knowledge, this is the first study to examine the alterations in global functional organization and connectivity in active CD patients based on fMRI. First, compared with heathy control, functional brain networks of active CD patients showed a significant increase in global efficiency. In addition, significant decreases in shortest path length and cluster efficiency in were found in active CD patients compared with NCs. Second, both active CD patients and healthy controls displayed small world topology in functional brain network, but active CD patients revealed significantly increased of small world organization than healthy control. Finally, we found significant reductions in rich club, feeder and local connections in active CD patients than NCs. Therefore, our results may provide new insights into understanding how hypercortisolism affects functional brain networks in active CD patients.

Functional MRI is an indirect measure of neural activity, by detecting the blood oxygen level and can be used to analyze activity of specific brain regions [19]. Functional MRI has been wildly used as a non-invasive brain imaging technique in the field of neuroscience [20]. Classic fMRI studies of task-related brain activation, which analyzes brain activity under specific experimental task conditions. In recent years, researchers have found that activation of brain during resting state play an important role in disease diagnosis. In this study, resting functional networks were used to investigate the correlations between time series in different brain regions, based on the effect of blood oxygen level. The correlation of different nodes (brain regions) can be analyzed with the help of graph theory, further the whole brain functional connections at in resting state were analyzed [21]. For active CD patients, it's quite different from other diseases that can cause brain atrophy, the functional brain networks were more interconnected than healthy control, which included increased global efficiency, decreased path length and decreased clustering coefficient. This phenomenon of increased interconnectivity has also been reported in other studies of traumatic brain injury and brain tumors [22, 23]. Karen et al. has put forth research findings traumatic brain injury show the increased local efficiency and connectivity degree compared with healthy controls, and suggested that these changes may reflect functional compensation [22]. Castellanos et al. reported that higher densely interconnectivity may be the result of higher cost consumption [24]. Changes in brain network connectivity can be influenced by the changes in hormone levels, and hormones can have complex influence on brain networks [25, 26]. Sripada et al. reported that dehydroepiandrosterone can shift the balance between default mode network and salience network [27]. Cushing’s disease provides a unique and naturalist model for studying the influence of hypercortisolism on brain function and structure [28]. Jiang et al. reported that active CD patients exhibited significantly altered diffuse parameters in the gray matter and white matter of the left medial temporal lobe and might explain some part of the memory and cognition impairments in active CD patients [4]. Additionally, the abnormal alterations in the amplitude of low-frequency fluctuation (ALFF) / regional homogeneity (ReHo) values in the posterior cingulate cortex (PCC) / precuneus (PCu) showed a significant correlation with cortisol levels based on functional MRI [29]. van der Werf et al. found abnormal increases in resting-state functional connectivity of long-term remission of active CD patients based on functional MRI [30]. The abnormal functional connectivity observed during our study of active CD patients may be due to hypercortisolism; however, the underlying mechanisms require further study.

Both Sporns et al. and Achard et al. confirmed that human brain has the small world properties and is characterized by high local clustering of connections between neighboring regions but with short path lengths between any pair of nodes [31, 32]. It's play an important role in achieving functional segregation and integration for complex brain networks [33]. The features of functional brain networks identified in our study for both active CD patients and healthy controls are consistent with small world network organization. However, changes between active CD patients and healthy controls were observed in this study. The normalized path lengths (λ) were low and showed significant differences between active CD and healthy control, which suggesting that it's conducive to rapid information exchange between spatially separated brain regions. This finding parallels results obtained with measures of shortest path length. The normalized cluster efficiency (γ) was increased and significant differences between active CD and healthy control, suggesting the ability of processing local information was enforced. Additionally, values for Sigma, was significantly higher in active CD compare with control group. These findings are in line with the Korenkevych et al's hypothesis that needs better brain network system to carry out normal everyday life for active CD patients [34]. These findings are consistent with other studies in different disease. Supekar at al. found abnormal changes of low normalized path lengths in small world organization for Alzheimer’s disease based on functional MRI [35]. Anand et al. indicated that abnormal small world organization may be associated with the cognitive impairments observed during traumatic brain injury [36].

In this study, we found rich club organization is presented in active CD patients but decreased compared with healthy control for the first time. Rich club organization is an important feature of brain network and abnormal changes has been found in other neurologic disease [37, 38]. The hub distributions of active CD patients were consistent with healthy control and other studies reported, but there is still some difference. One possible explanation for this is the differential distribution of glucocorticoids in brain. Despite rich club paly a high role in information exchange between different regions, it's vulnerable to attack [39, 40]. Previous studies revealed that the impact of alterations of rich club connection can be compensated by increasing local connections. However, we found that connections of rich club, feeder and local regions were decreased in active CD patients. It means that widespread disruption of gray matter connectivity. One possible reason for this is that glucocorticoid receptors are widely distributed in our brain [41]. This was corroborated by numerous studies that volume of grey matter in active CD patients was reduced for hypercortisolism [42–44]. Abnormal changes of rich club organization have also been found in other neuropsychiatric diseases. In patients with subjective cognitive decline, both hub and local region connections showed lower strength compared with healthy control and have relationships with auditory verbal learning test [45]. In schizophrenia patients, the reduced rich club connection was associated with cognitive decline [46].

We performed a correlation analysis between clinical information and network parameters and found no correlations between disease duration, ACTH levels, and brain network parameters. The lack of correlations may be due to the small sample size used in this study, which may have introduced bias. Therefore, whether ACTH and disease duration can effectively reflect the severity of CD remains controversial.

Our study has some limitations. First, the sample size is relatively small, but consistent with similar studies investigating topological parameters [47–49]. It's hard to recruit large samples of active CD patients for it is a rare disease [1]. Second, we did not investigate the correlation between CD patients and topological organizations and it needs further investigation.

In summary, we showed that functional brain networks were abnormal changed in active CD patients by applying topological analysis based on resting fMRI. Our study revealed the abnormal changes of small world and rich club organization of active CD patients. Although we didn’t find significant correlation between the severity of CD and the changes of the parameters, we will continue relevant research in the future study**.** Graph theoretical analysis provide us new insight into understanding the effect of active CD on our brain.

## MATERIALS AND METHODS

### Participants

Nineteen active CD patients and twenty-two age and education matched healthy controls (NC) were included in our study. Disease duration was recorded from first symptom onset as previously reported [50]. Nineteen active CD patients were performed transsphenoidal surgery. Eligibility criteria for the study were (a) 18~60 years of age, (b) positive pituitary lesions in imaging examination. Exclusion criteria included a history of drug or alcohol abuse, history of traumatic brain injury, neurological problems, contraindications for undergoing a magnetic resonance imaging scan and left-handedness.

Following the 2008 Endocrine Society guidelines, Cushing’s disease and its etiology were confirmed by clinical features (e.g., truncal obesity, skin and muscle atrophy, and moon face), elevated 24-hour urinary free GC (UFC), absence of blunted circadian rhythm of GC secretion, elevated ACTH levels, lack of suppression after low dose dexamethasone (2 mg) administration, 50% suppression after high dose dexamethasone (8 mg) administration, a central to peripheral (C/P) ACTH ratio ≥2 for bilateral petrosal sinus sampling (BIPSS) and pathology after surgery [51]. All aCD patients were treated with transsphenoidal surgery by same doctor and without radiotherapy or other surgery treatment as we have been previously reported [29]. All active patients were confirmed in our hospital by surgical pathological findings. They did not receive any other systematic therapy in other hospitals. The direct chemiluminescence immunoassays were used to determine the level of ACTH, serum cortisol, and 24UFC.

Biometric measurements of all the active CD patients were collected, including 24-hour urinary free GC (UFC) levels and adrenocorticotropin (ACTH) levels from a peripheral vein. The medical history and medication use of all the study subjects were recorded according to a standardized questionnaire.

### Image acquisition

All the subjects were scanned using a 3.0T MRI scanner (GE Signa Excite HD; GE Medical Systems, Milwaukee, WI, USA) with a birdcage head coil. MRI protocol include T1-weighted sequence images were acquired: TR = 5.576 ms; TE = 1.752 ms; slices = 196; thickness = 1 mm; gap = 0 mm; FA = 908; acquisition matrix = 256×256; and FOV = 250 mm×250 mm. For resting-state imaging: petition time (TR) = 2000 ms; echo time (TE) = 30 ms; slices = 35; thickness = 4 mm; gap = 0 mm; field of view (FOV) = 240 mm×240 mm; acquisition matrix = 64×64; and flip angle (FA) = 90°. Participants were instructed to close their eyes and relax during rest but stay awake while avoiding any structured thinking. No specific cognitive task was given. Imaging data for all patients were completed within three days before surgery.

### Image processing

**Figure 6:**
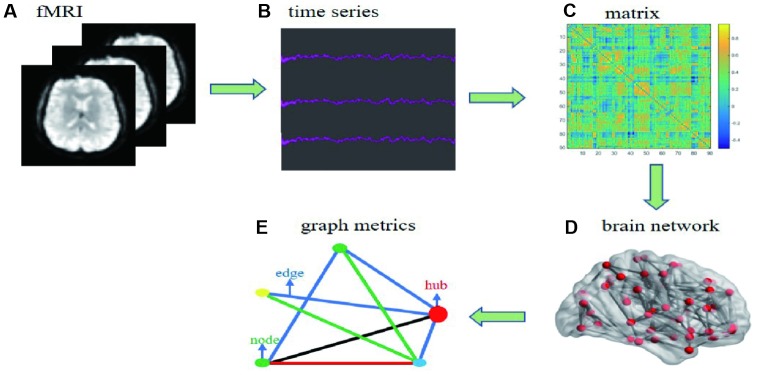
**Flow chart of date processing for resting functional MRI.** (**A**) individual fMRI images were used for parceling the distinct brain regions. (**B**) time series were collected after the pretreatment based on bold oxygenation level dependent. (**C**) functional connectivity matrix between node i and j was constructed. (**D**) individual brain network was collected. (**E**) simple model diagram for graph theory analysis.

Images were processed with Statistical Parametric Mapping software (SPM12 Wellcome Department, University College London, London, England) implemented in MATLAB (version R2014b; MathWorks, Natick, MA). The first 10 volumes were discarded for magnetization, leaving 200 images available for analysis. Slice-timing and realignment were performed to correct for head motion and two subjects (1 CD patient and 1 NC) were excluded for the excessive head motion (> 3mm and 3°). The images were then normalized to Montreal Neurological Institute (MNI) EPI template and resampled to a 3-mm cubic voxel. Images were further smoothing with an 4mm full-width at half maximum (FWHM) isotropic Gaussian kernel. Finally, linear drift and temporal band-pass filtering (0.01<f<0.08) were removed to reduce the effects of low-frequency drift and high-frequency noise (Figure 6). The results were visually checked for each participant by an experienced neuroscientist.

### Network construction

Brain network includes nodes and edges. In this study, we use automated anatomic labeling template 90 (AAL 90) to define network nodes [52]. The Pearson correlation coefficients between any two areas of 90 nodes were defined to network edges. Finally, the binary 90*90 functional connectivity matrix was constructed for each participant. A series of threshold of sparsity were set to assess the effects of thresholds ranging from 0.05 to 0.4 at interval of 0.01 [53], which removed spurious edges as much as possible (Figure 6).

### Graph metrics

Graph metrics were analyzed by using Gretna and viewed by BrainNet Viewer software [54]. In this study, we calculated the global efficiency (Eglo), local efficiency (Eloc), clustering coefficients (Cp), shortest path length (Lp), small-world parameters, and rich-club parameters. Global efficiency reflected the efficiency of the parallel information in the whole network. Local efficiency reveals how much the efficient between the first neighbors of each node, it reflects ability to resist external attacks of brain network. Shortest path length of a network indicated the ability for information to propagate in parallel. Cluster coefficient means the possibility of whether the neighborhoods were linked with each other and indicates the local interconnectivity in the in the whole network. (More information can be seen in the Supplementary Material).

### Small world

In this study, we computed the small-world organization of the binary network of all participants. The small-world network reals that it has higher local interconnectivity approximately equivalent shortest path length compared with random network [55, 56]. The construction of small-world networks is the best balance between simultaneous specialization and integration of function [57]. (More information can be seen in the Supplementary Material).

### Rich club

According to the graph theory, node can be organized into rich-club and peripheral nodes. Hubs regions were defined as the highly connected and central brain regions (nodes), its more densely interconnected, which called rich club pheromone, than random networks [56, 58, 59]. It plays a high role in guiding function controlling integration and information flow in the brain network [60]. Local region was defined as regions other than hubs. In this study, the degree centrality, was used to exam the nodal characteristics of each brain region in functional brain network. The hub regions were defined with a degree centrality at least 1 standard deviation above the mean degree centrality across all regions [8, 61]. Furthermore, we calculated the rich club connections, feeder connections and local connections of each group respectively. (More information can be seen in the Supplementary Material).

### Statistical analysis

Statistical analysis was performed in SPSS software (version 22.0; Inc., Chicago, IL). Differences in gender distribution between two groups were determined using a chi-square test. Differences in age and education level between two groups were determined by between-group t-tests for means. Network matrices (network efficiency, cluster efficiency and path length) between two groups were compared by using two-sample t-test. A value of p < 0.05 was considered to be significant. We calculated spearman correlations between network parameters and clinical parameters, including ACTH and disease duration. We used permutation test (100 permutations) to calculate the group difference about rich club connection strength between CD patients and healthy control. We selected false discovery rate (FDR) to 1% to protect against type I errors when performing multiple comparisons.

## Supplementary Material

Supplementary Materials

## References

[1] Newell-Price J, Bertagna X, Grossman AB, Nieman LK. Cushing’s syndrome. Lancet. 2006; 367:1605–17. 10.1016/S0140-6736(06)68699-6 16698415

[2] Simmons NE, Do HM, Lipper MH, Laws ER Jr. Cerebral atrophy in Cushing’s disease. Surg Neurol. 2000; 53:72–76. 10.1016/S0090-3019(99)00197-4 10697236

[3] Khiat A, Bard C, Lacroix A, Boulanger Y. Recovery of the brain choline level in treated Cushing’s patients as monitored by proton magnetic resonance spectroscopy. Brain Res. 2000; 862:301–07. 10.1016/S0006-8993(00)02147-8 10799704

[4] Jiang H, He NY, Sun YH, Jian FF, Bian LG, Shen JK, Yan FH, Pan SJ, Sun QF. Altered gray and white matter microstructure in Cushing’s disease: A diffusional kurtosis imaging study. Brain Res. 2017; 1665:80–87. 10.1016/j.brainres.2017.04.007 28438531

[5] Pivonello R, Simeoli C, De Martino MC, Cozzolino A, De Leo M, Iacuaniello D, Pivonello C, Negri M, Pellecchia MT, Iasevoli F, Colao A. Neuropsychiatric disorders in Cushing's syndrome. Front Neurosci. 2015; 9:129. 10.3389/fnins.2015.00129 25941467 PMC4403344

[6] Rubinov M, Sporns O. Complex network measures of brain connectivity: uses and interpretations. Neuroimage. 2010; 52:1059–69. 10.1016/j.neuroimage.2009.10.003 19819337

[7] Hagmann P, Kurant M, Gigandet X, Thiran P, Wedeen VJ, Meuli R, Thiran JP. Mapping human whole-brain structural networks with diffusion MRI. PLoS One. 2007; 2:e597. 10.1371/journal.pone.0000597 17611629 PMC1895920

[8] Shu N, Wang X, Bi Q, Zhao T, Han Y. Disrupted Topologic Efficiency of White Matter Structural Connectome in Individuals with Subjective Cognitive Decline. Radiology. 2018; 286:229–38. 10.1148/radiol.2017162696 28799862

[9] Stellmann JP, Hodecker S, Cheng B, Wanke N, Young KL, Hilgetag C, Gerloff C, Heesen C, Thomalla G, Siemonsen S. Reduced rich-club connectivity is related to disability in primary progressive MS. Neurol Neuroimmunol Neuroinflamm. 2017; 4:e375. 10.1212/NXI.0000000000000375 28804744 PMC5532749

[10] Hall JM, Shine JM, Ehgoetz Martens KA, Gilat M, Broadhouse KM, Szeto JY, Walton CC, Moustafa AA, Lewis SJ. Alterations in white matter network topology contribute to freezing of gait in Parkinson’s disease. J Neurol. 2018; 265:1353–64. 10.1007/s00415-018-8846-3 29616302

[11] Koenis MMG, Brouwer RM, Swagerman SC, van Soelen ILC, Boomsma DI, Hulshoff Pol HE. Association between structural brain network efficiency and intelligence increases during adolescence. Hum Brain Mapp. 2018; 39:822–36. 10.1002/hbm.23885 29139172 PMC6866576

[12] Oldham S, Fornito A. The development of brain network hubs. Dev Cogn Neurosci. 2019; 36:100607. 10.1016/j.dcn.2018.12.005 30579789 PMC6969262

[13] van den Heuvel MP, Kahn RS, Goñi J, Sporns O. High-cost, high-capacity backbone for global brain communication. Proc Natl Acad Sci USA. 2012; 109:11372–7. 10.1073/pnas.1203593109 22711833 PMC3396547

[14] Iraji A, Benson RR, Welch RD, O’Neil BJ, Woodard JL, Ayaz SI, Kulek A, Mika V, Medado P, Soltanian-Zadeh H, Liu T, Haacke EM, Kou Z. Resting State Functional Connectivity in Mild Traumatic Brain Injury at the Acute Stage: Independent Component and Seed-Based Analyses. J Neurotrauma. 2015; 32:1031–45. 10.1089/neu.2014.3610 25285363 PMC4504339

[15] Pires P, Santos A, Vives-Gilabert Y, Webb SM, Sainz-Ruiz A, Resmini E, Crespo I, de Juan-Delago M, Gómez-Anson B. White matter alterations in the brains of patients with active, remitted, and cured cushing syndrome: a DTI study. AJNR Am J Neuroradiol. 2015; 36:1043–48. 10.3174/ajnr.A4322 25929879 PMC8013026

[16] Dettwiler A, Murugavel M, Putukian M, Cubon V, Furtado J, Osherson D. Persistent differences in patterns of brain activation after sports-related concussion: a longitudinal functional magnetic resonance imaging study. J Neurotrauma. 2014; 31:180–88. 10.1089/neu.2013.2983 23914845 PMC3900041

[17] Johnson B, Zhang K, Gay M, Horovitz S, Hallett M, Sebastianelli W, Slobounov S. Alteration of brain default network in subacute phase of injury in concussed individuals: resting-state fMRI study. Neuroimage. 2012; 59:511–18. 10.1016/j.neuroimage.2011.07.081 21846504 PMC3196274

[18] Mayer AR, Mannell MV, Ling J, Gasparovic C, Yeo RA. Functional connectivity in mild traumatic brain injury. Hum Brain Mapp. 2011; 32:1825–35. 10.1002/hbm.21151 21259381 PMC3204375

[19] Sharp DJ, Scott G, Leech R. Network dysfunction after traumatic brain injury. Nat Rev Neurol. 2014; 10:156–66. 10.1038/nrneurol.2014.15 24514870

[20] Sporns O. Contributions and challenges for network models in cognitive neuroscience. Nat Neurosci. 2014; 17:652–60. 10.1038/nn.3690 24686784

[21] Sporns O, Tononi G, Kötter R. The human connectome: A structural description of the human brain. PLoS Comput Biol. 2005; 1:e42. 10.1371/journal.pcbi.0010042 16201007 PMC1239902

[22] Caeyenberghs K, Leemans A, Heitger MH, Leunissen I, Dhollander T, Sunaert S, Dupont P, Swinnen SP. Graph analysis of functional brain networks for cognitive control of action in traumatic brain injury. Brain. 2012; 135:1293–307. 10.1093/brain/aws048 22427332

[23] Bartolomei F, Bosma I, Klein M, Baayen JC, Reijneveld JC, Postma TJ, Heimans JJ, van Dijk BW, de Munck JC, de Jongh A, Cover KS, Stam CJ. Disturbed functional connectivity in brain tumour patients: evaluation by graph analysis of synchronization matrices. Clin Neurophysiol. 2006; 117:2039–49. 10.1016/j.clinph.2006.05.018 16859985

[24] Castellanos NP, Leyva I, Buldú JM, Bajo R, Paúl N, Cuesta P, Ordóñez VE, Pascua CL, Boccaletti S, Maestú F, del-Pozo F. Principles of recovery from traumatic brain injury: reorganization of functional networks. Neuroimage. 2011; 55:1189–99. 10.1016/j.neuroimage.2010.12.046 21195199

[25] Andreano JM, Touroutoglou A, Dickerson B, Barrett LF. Hormonal Cycles, Brain Network Connectivity, and Windows of Vulnerability to Affective Disorder. Trends Neurosci. 2018; 41:660–76. 10.1016/j.tins.2018.08.007 30274602 PMC6481680

[26] Williams LM. Defining biotypes for depression and anxiety based on large-scale circuit dysfunction: a theoretical review of the evidence and future directions for clinical translation. Depress Anxiety. 2017; 34:9–24. 10.1002/da.22556 27653321 PMC5702265

[27] Sripada RK, Welsh RC, Marx CE, Liberzon I. The neurosteroids allopregnanolone and dehydroepiandrosterone modulate resting-state amygdala connectivity. Hum Brain Mapp. 2014; 35:3249–61. 10.1002/hbm.22399 24302681 PMC4739102

[28] Toffanin T, Nifosì F, Follador H, Passamani A, Zonta F, Ferri G, Scanarini M, Amistà P, Pigato G, Scaroni C, Mantero F, Carollo C, Perini GI. Volumetric MRI analysis of hippocampal subregions in Cushing’s disease: a model for glucocorticoid neural modulation. Eur Psychiatry. 2011; 26:64–67. 10.1016/j.eurpsy.2010.09.003 21067899

[29] Jiang H, He NY, Sun YH, Jian FF, Bian LG, Shen JK, Yan FH, Pan SJ, Sun QF. Altered spontaneous brain activity in Cushing’s disease: a resting-state functional MRI study. Clin Endocrinol (Oxf). 2017; 86:367–76. 10.1111/cen.13277 27859451

[30] van der Werff SJ, Pannekoek JN, Andela CD, Meijer OC, van Buchem MA, Rombouts SA, van der Mast RC, Biermasz NR, Pereira AM, van der Wee NJ. Resting-State Functional Connectivity in Patients with Long-Term Remission of Cushing’s Disease. Neuropsychopharmacology. 2015; 40:1888–98. 10.1038/npp.2015.38 25652248 PMC4839512

[31] Sporns O, Honey CJ. Small worlds inside big brains. Proc Natl Acad Sci USA. 2006; 103:19219–20. 10.1073/pnas.0609523103 17159140 PMC1748207

[32] Achard S, Bullmore E. Efficiency and cost of economical brain functional networks. PLoS Comput Biol. 2007; 3:e17. 10.1371/journal.pcbi.0030017 17274684 PMC1794324

[33] Bassett DS, Bullmore ET. Small-World Brain Networks Revisited. Neuroscientist. 2017; 23:499–516. 10.1177/1073858416667720 27655008 PMC5603984

[34] Korenkevych D, Chien JH, Zhang J, Shiau DS, Sackellares C, Pardalos PM. Small World Networks in Computational Neuroscience. In: Pardalos PM, Du DZ, Graham RL, editors. Handbook of Combinatorial Optimization. New York (NY): Springer New York; 2013 pp. 3057–88. 10.1007/978-1-4419-7997-1_70

[35] Supekar K, Menon V, Rubin D, Musen M, Greicius MD. Network analysis of intrinsic functional brain connectivity in Alzheimer’s disease. PLoS Comput Biol. 2008; 4:e1000100. 10.1371/journal.pcbi.1000100 18584043 PMC2435273

[36] Pandit AS, Expert P, Lambiotte R, Bonnelle V, Leech R, Turkheimer FE, Sharp DJ. Traumatic brain injury impairs small-world topology. Neurology. 2013; 80:1826–33. 10.1212/WNL.0b013e3182929f38 23596068 PMC3908350

[37] van den Heuvel MP, Sporns O. Rich-club organization of the human connectome. J Neurosci. 2011; 31:15775–86. 10.1523/JNEUROSCI.3539-11.2011 22049421 PMC6623027

[38] Griffa A, Van den Heuvel MP. Rich-club neurocircuitry: function, evolution, and vulnerability. Dialogues Clin Neurosci. 2018; 20:121–32. 30250389 10.31887/DCNS.2018.20.2/agriffa PMC6136122

[39] Crossley NA, Mechelli A, Scott J, Carletti F, Fox PT, McGuire P, Bullmore ET. The hubs of the human connectome are generally implicated in the anatomy of brain disorders. Brain. 2014; 137:2382–95. 10.1093/brain/awu132 25057133 PMC4107735

[40] Stam CJ. Modern network science of neurological disorders. Nat Rev Neurosci. 2014; 15:683–95. 10.1038/nrn3801 25186238

[41] Pereira AM, Tiemensma J, Romijn JA. Neuropsychiatric disorders in Cushing’s syndrome. Neuroendocrinology. 2010 (Suppl 1); 92:65–70. 10.1159/000314317 20829621

[42] Santos A, Resmini E, Crespo I, Pires P, Vives-Gilabert Y, Granell E, Valassi E, Gómez-Anson B, Martínez-Momblán MA, Mataró M, Webb SM. Small cerebellar cortex volume in patients with active Cushing’s syndrome. Eur J Endocrinol. 2014; 171:461–69. 10.1530/EJE-14-0371 25005936

[43] Starkman MN, Gebarski SS, Berent S, Schteingart DE. Hippocampal formation volume, memory dysfunction, and cortisol levels in patients with Cushing’s syndrome. Biol Psychiatry. 1992; 32:756–65. 10.1016/0006-3223(92)90079-F 1450290

[44] Bourdeau I, Bard C, Noël B, Leclerc I, Cordeau MP, Bélair M, Lesage J, Lafontaine L, Lacroix A. Loss of brain volume in endogenous Cushing’s syndrome and its reversibility after correction of hypercortisolism. J Clin Endocrinol Metab. 2002; 87:1949–54. 10.1210/jcem.87.5.8493 11994323

[45] Shu N, Liang Y, Li H, Zhang J, Li X, Wang L, He Y, Wang Y, Zhang Z. Disrupted topological organization in white matter structural networks in amnestic mild cognitive impairment: relationship to subtype. Radiology. 2012; 265:518–27. 10.1148/radiol.12112361 22984189

[46] Collin G, de Nijs J, Hulshoff Pol HE, Cahn W, van den Heuvel MP. Connectome organization is related to longitudinal changes in general functioning, symptoms and IQ in chronic schizophrenia. Schizophr Res. 2016; 173:166–73. 10.1016/j.schres.2015.03.012 25843919

[47] Mai N, Zhong X, Chen B, Peng Q, Wu Z, Zhang W, Ouyang C, Ning Y. Weight Rich-Club Analysis in the White Matter Network of Late-Life Depression with Memory Deficits. Front Aging Neurosci. 2017; 9:279. 10.3389/fnagi.2017.00279 28878666 PMC5572942

[48] van der Horn HJ, Scheenen ME, de Koning ME, Liemburg EJ, Spikman JM, van der Naalt J. The Default Mode Network as a Biomarker of Persistent Complaints after Mild Traumatic Brain Injury: A Longitudinal Functional Magnetic Resonance Imaging Study. J Neurotrauma. 2017; 34:3262–69. 10.1089/neu.2017.5185 28882089

[49] Verhelst H, Vander Linden C, De Pauw T, Vingerhoets G, Caeyenberghs K. Impaired rich club and increased local connectivity in children with traumatic brain injury: Local support for the rich? Hum Brain Mapp. 2018; 39:2800–11. 10.1002/hbm.24041 29528158 PMC6866640

[50] Resmini E, Santos A, Gómez-Anson B, Vives Y, Pires P, Crespo I, Portella MJ, de Juan-Delago M, Barahona MJ, Webb SM. Verbal and visual memory performance and hippocampal volumes, measured by 3-Tesla magnetic resonance imaging, in patients with Cushing’s syndrome. J Clin Endocrinol Metab. 2012; 97:663–71. 10.1210/jc.2011-2231 22162471

[51] Biller BM, Grossman AB, Stewart PM, Melmed S, Bertagna X, Bertherat J, Buchfelder M, Colao A, Hermus AR, Hofland LJ, Klibanski A, Lacroix A, Lindsay JR, et al. Treatment of adrenocorticotropin-dependent Cushing’s syndrome: a consensus statement. J Clin Endocrinol Metab. 2008; 93:2454–62. 10.1210/jc.2007-2734 18413427 PMC3214276

[52] Tzourio-Mazoyer N, Landeau B, Papathanassiou D, Crivello F, Etard O, Delcroix N, Mazoyer B, Joliot M. Automated anatomical labeling of activations in SPM using a macroscopic anatomical parcellation of the MNI MRI single-subject brain. Neuroimage. 2002; 15:273–89. 10.1006/nimg.2001.0978 11771995

[53] Fang J, Chen H, Cao Z, Jiang Y, Ma L, Ma H, Feng T. Impaired brain network architecture in newly diagnosed Parkinson’s disease based on graph theoretical analysis. Neurosci Lett. 2017; 657:151–58. 10.1016/j.neulet.2017.08.002 28789983

[54] Wang J, Wang X, Xia M, Liao X, Evans A, He Y. GRETNA: a graph theoretical network analysis toolbox for imaging connectomics. Front Hum Neurosci. 2015; 9:386. 10.3389/fnhum.2015.00386 26175682 PMC4485071

[55] Bassett DS, Bullmore ET. Human brain networks in health and disease. Curr Opin Neurol. 2009; 22:340–47. 10.1097/WCO.0b013e32832d93dd 19494774 PMC2902726

[56] van den Heuvel MP, Stam CJ, Boersma M, Hulshoff Pol HE. Small-world and scale-free organization of voxel-based resting-state functional connectivity in the human brain. Neuroimage. 2008; 43:528–39. 10.1016/j.neuroimage.2008.08.010 18786642

[57] Sporns O, Zwi JD. The small world of the cerebral cortex. Neuroinformatics. 2004; 2:145–62. 10.1385/NI:2:2:145 15319512

[58] Hagmann P, Cammoun L, Gigandet X, Meuli R, Honey CJ, Wedeen VJ, Sporns O. Mapping the structural core of human cerebral cortex. PLoS Biol. 2008; 6:e159. 10.1371/journal.pbio.0060159 18597554 PMC2443193

[59] Sporns O, Honey CJ, Kötter R. Identification and classification of hubs in brain networks. PLoS One. 2007; 2:e1049. 10.1371/journal.pone.0001049 17940613 PMC2013941

[60] Wang B, Zhan Q, Yan T, Imtiaz S, Xiang J, Niu Y, Liu M, Wang G, Cao R, Li D. Hemisphere and Gender Differences in the Rich-Club Organization of Structural Networks. Cereb Cortex. 2019; 29:4889–901. 10.1093/cercor/bhz027 30810159

[61] Lo CY, Wang PN, Chou KH, Wang J, He Y, Lin CP. Diffusion tensor tractography reveals abnormal topological organization in structural cortical networks in Alzheimer’s disease. J Neurosci. 2010; 30:16876–85. 10.1523/JNEUROSCI.4136-10.2010 21159959 PMC6634928

