# Supplementary material for: Impaired brain network architecture in Cushing’s disease based on graph theoretical analysis
Source: Aging (Albany NY). 2020 Mar 24;12(6):5168–82. doi: 10.18632/aging.102939 (PMC7138581; doi:10.18632/aging.102939)
Supplement: Supplementary Materials [file aging-12-102939-s001..pdf]

SUPPLEMENTARY MATERIALS

The basic approach to analyze complex system (information connection between brain regions) named as graph theoretical [1]. The basic elements of network are nodes (brain regions) and edges (connectivity between nodes). Functional brain networks can be quantitatively described with help of graph theory by calculating a variety of organizations [2]. In this study, wo focus on whole brain network which reflect the brain activity and function connectivity by calculating network organization [3].

Definitions of network organization

In this study, we calculated the global efficiency (Eglob), local efficiency (Eloc), shortest path length (Lp), cluster efficiency (Cp). All network organizations were quantified using the GRETNA software (http://www.nitrc.org/projects/gretna/) and viewed by using the BrainNet Viewer software (http://www.nitrc.org/projects/bnv/).

Global efficiency (Eglob)

Global efficiency
Global efficiency reflects the ability of information transmission in a network [4].

For a network G, the equitation is defined as:

$$\text{Eglob}\,(\text{G}) = \frac{1}{N(N-1)} \sum i \neq j \in G \frac{1}{Lij}$$

Where the Lij is the shortest path length between node i and node j in G.

Local efficiency

The local efficiency of G measures the how much of the network is fault tolerant and reveals how efficient the communication is among the first neighbors of the node i when it is removed [5]. For a network G, the equitation is defined as:

$$\text{Eloc(G)} = \frac{1}{N} \sum\nolimits_{i \in G} \text{Eglob}(\text{G}_i)$$

Where the G_i is the subnetwork composed of the nearest neighbors of node i.

Shortest path length

The shortest path length
The shortest path length is defined as the shortest edge between node i and node j.

The average of all shortest lengths between each pair of nodes in the network is global defined as global shortest path length. For a network G, the equitation is defined as:

$$\text{Lp(G)} = \frac{1}{N(\text{N}-1)} \sum\nolimits_{i \neq j \in G} Lij$$

Where Lij is the shortest path length between node i and node j. N=90.

Cluster efficiency

The cluster efficiency of node i is defined as the likelihood of neighbor to neighbor connection. The global cluster efficiency is the average of the cluster efficiency overall nodes and reveled the larger extent of the local interconnectivity of a network. For a network G, the equitation is defined as:

$$\text{Cp} = \frac{1}{N} \times \sum \left\{ \sum\nolimits_{i \in G} \left[{}^{2}\!/\!_{k_i} (k_i)(k_i - 1) \sum\nolimits_{j,k} \left(\omega_{ij} \omega_{jk} \omega_{ki} \right)^{\frac{1}{3}} \right] \right\}$$

Where K_i the degree of node i and ω_{ij} is the weight between node i and node j. N = 90.

Small world

In this study, we calculated the small world properties of the binary functional brain networks. Small world organization include normalized global shortest path length, normalized global clustering and small-world ness. 100 random networks were generated before calculated small world organization, which have the same numbers of nodes and edges as the real network [6]. The normalized global shortest path length (Lambda)=Lp^{real}/Lp^{rand}, global normalized global clustering (Gamma)=Cp^{real}/Cp^{rand}, small worldness (Sigma)= Lambda/Gamma. Where Lp^{rand} and Cp^{rand} are the means of 100 random network global clustering coefficients and the global shortest path length, respectively. If the Sigam>1 or Lambda>1 and Gamma=1, we can say the network existence of small world orgnazation [7].

Rich club

The phenomenon of rich club means that the hubs were densely connect to each other regions in brain network [8]. It plays a vital role in exchanging information in the brain network. However, rich club organization may be vulnerable to brain stress, such as traumatic brain injury and AD, for high connectivity density and metabolic demand [9]. In this study, we constructed the functional

brain network and identified the brain hubs. In this study, the degree centrality, was used to exam the nodal characteristics of each brain region in functional brain network. The hub regions were defined with a degree centrality at least 1 standard deviation above the mean degree centrality across all regions [10]. Local region was defined as regions other than hubs.

REFERENCES

1. Rubinov M, Sporns O. Complex network measures of brain connectivity: uses and interpretations. Neuroimage. 2010; 52:1059–69. https://doi.org/10.1016/j.neuroimage.2009.10.003 PMID:19819337

2. Bullmore E, Sporns O. Complex brain networks: graph theoretical analysis of structural and functional systems. Nat Rev Neurosci. 2009; 10:186–98. https://doi.org/10.1038/nrn2575 PMID:19190637

3. Sharp DJ, Scott G, Leech R. Network dysfunction after traumatic brain injury. Nat Rev Neurol. 2014; 10:156–66. https://doi.org/10.1038/nrneurol.2014.15 PMID:24514870

4. Latora V, Marchiori M. Efficient behavior of small-world networks. Phys Rev Lett. 2001; 87:198701. https://doi.org/10.1103/PhysRevLett.87.198701 PMID:11690461

5. Shu N, Liang Y, Li H, Zhang J, Li X, Wang L, He Y, Wang Y, Zhang Z. Disrupted topological organization in white matter structural networks in amnestic mild cognitive impairment: relationship to subtype. Radiology. 2012; 265:518–27. https://doi.org/10.1148/radiol.12112361 PMID:22984189

6. Maslov S, Sneppen K. Specificity and stability in topology of protein networks. Science. 2002; 296:910–13. https://doi.org/10.1126/science.1065103 PMID:11988575

7. Watts DJ, Strogatz SH. Collective dynamics of ‘small-world’ networks. Nature. 1998; 393:440–42. https://doi.org/10.1038/30918 PMID:9623998

8. Stellmann JP, Hodecker S, Cheng B, Wanke N, Young KL, Hilgetag C, Gerloff C, Heesen C, Thomalla G, Siemonsen S. Reduced rich-club connectivity is related to disability in primary progressive MS. Neurol Neuroimmunol Neuroinflamm. 2017; 4:e375. https://doi.org/10.1212/NXI.0000000000000375 PMID:28804744

9. Stam CJ. Modern network science of neurological disorders. Nat Rev Neurosci. 2014; 15:683–95. https://doi.org/10.1038/nrn3801 PMID:25186238

10. Shu N, Wang X, Bi Q, Zhao T, Han Y. Disrupted Topologic Efficiency of White Matter Structural Connectome in Individuals with Subjective Cognitive Decline. Radiology. 2018; 286:229–38. https://doi.org/10.1148/radiol.2017162696 PMID:28799862
